# Supra-Pubic vs. Perineal Lithotomy

**Published:** 1886-06

**Authors:** W. S. Tremaine

**Affiliations:** Major and Surgeon, U. S. A.; Surgeon-in-Chief, Sisters of Charity Hospital, Buffalo, N. Y.


### Selections.

### Supra-Pubic vs. Perineal Lithotomy.

By W. S. Tremaine, M. D.,

Major and Surgeon, U. S. A.; Surgeon-in-Chief, Sisters of Charity Hospital, Buffalo, N. Y.

*To the Editor of the Lancet:*

Sir,—In *The Lancet* of Jan. 30, 1886, there is reference made to a case of attempted supra-pubic lithotomy by M. Polaillon, which was abandoned on account of anomalous distribution of the peritoneum. It is difficult to understand why a supra-pubic operation was attempted to remove a "stone measuring in circumference four centimeters by two"; stranger yet that it should have been abandoned through dread of wounding the peritoneum; as this may occur without disaster, the following case of epi-cystotomy will illustrate:

John M———, a native of Ireland, aged seventy-three, was admitted to the Buffalo Hospital of the Sisters of Charity, on Sept. 22, 1884, with a history of symptoms of stone for the past two years. During the three months prior to admission to the hospital he had suffered intense pain, with cystitis. Examination with the sound revealed the presence of stone in the bladder, greatly enlarged prostate, and sub-acute cystitis.

On Sept. 23, 1884, in the presence of a large number of medical gentlemen, and with the assistance of my colleagues, Professors Cronyn and Stockton, and Drs. Mickle and Clark, I performed epi-cystotomy. An effort was made to use Peterson's method of throwing the bladder upwards and forwards by introducing a rubber bag into the rectum and inflating it; but owing to an imperfect apparatus and the excessively enlarged prostate, the attempt was abandoned. The bladder was then injected with about six ounces of a solution of boro-glyceride (all it would hold). The patient took the anæsthetic (ether) badly. There was a constant convulsive movement of the abdominal muscles, and it seemed impossible to produce relaxation. This greatly complicated the operation, and partly accounts for the accident which occurred. The bladder was exposed by the usual incision, and seized by a pair of tissue forceps, which, by the way, are inferior for their purpose to the tenaculum. It was then incised, and a mulberry calculus, 1⅜ in. in long diameter by 1 in. transversely, extracted with the thumb and forefinger. After washing the bladder out with mercuric bichloride solution (1 in 1,000), a short rubber drainage-tube was inserted at the lower angle of the wound. The abdominal incision was then closed by silver-wire sutures, with the exception of about half an inch left open for drainage.

About fifteen minutes after the patient had been removed to his bed I was summoned in haste, and found, on reaching him, that he had vomited while recovering from the ether, and that a coil of small intestine about the size of the fist protruded from the wound. The stitches were rapidly cut, the intestine completely returned, and by means of a perineum needle four silver-wire sutures were carried through the abdominal walls and the anterior wall of the bladder; then anchoring the bladder to the abdominal wall, a soft rubber catheter was placed in the bladder per urethram, and a pad of gauze containing wood flour mixed with mercuric bichloride and naphthalin was applied over the wound.

Sept. 25th (8 P. M.): Temperature 101°; some pain in abdomen. Ordered one grain of powdered opium and two grains of camphor powder every four hours.—26th: Slept fairly; temperature 100°; bloody urine flowing freely through the catheter.—27th: Temperature normal; patient feeling well; urine clearer. —28th: Temperature normal; slept well; slight escape of very ammoniacal urine through wound; bladder washed out with boro-glyceride solution.—30th: Bowels fairly moved in response to a cathartic.—Oct. 1st: Temperature normal; bladder washed out with mercuric bichloride solution (1 in 1,000). From this time the patient steadily improved, the urine becoming clearer, and the wound healing completely. He was discharged cured on Oct. 23, 1884. Three weeks later he reported himself to Dr. Mickle at the Emergency Hospital, and stated that he was "quite well, and had no further trouble with his urinary organs."

Notwithstanding the peritoneum was wounded during the operation, the patient made an excellent recovery, without peritonitis or any other untoward symptom. From his advanced age and the complications above mentioned, this case was fairly a crucial test of the operation of epi-cystotomy, and goes far in support of the claim I have made for it in a previous paper. I suggest that an effective method in dealing with such an exceptional case as reported by M. Polaillon would be to pass through the abdominal wall and the anterior wall of the bladder a long steel pin, thus fastening the peritoneal surface of the bladder to that of the abdominal parietes, and, either before or after incising the peritoneum, to stitch with fine catgut sutures the vesical layer of peritoneum to that lining the abdominal walls, in this manner preventing the escape of urine into the peritoneal cavity. The fact that the bladder in M. Polaillon's case was diseased would suggest the propriety of inserting a tube into the bladder at the lower portion of the wound for thorough drainage. The impunity with which exploratory incisions are now made in cases of abdominal tumors lends strength to this method.

The advantages of the supra-pubic operation I had the honor to point out in a paper read before the American Surgical Association at its meeting in Washington, D. C., held April 30th to May 3d, 1884, in which I made use of the following language: "As regards the relative merits of epi-cystotomy and litholapaxy, recently discussed by Dittel, in properly selected cases and in experienced and skillful hands, the former will probably maintain its present position, but my own opinion is that supra-pubic cystotomy will prove by far the safer operation in the hands of the average surgeon." A more extensive experience in this class of cases has but served to confirm me in this position. It is gratifying to find my views endorsed by so eminent an authority as Sir Henry Thompson in *The Lancet* for July 25th, 1885, more than a year afterwards, where he says: "The problem, then, left for solution is, what is the best cutting operation for hard calculi (urates and oxalates) which weigh from about two ounces and upwards, as well as for those not quite so large, which are so peculiar in form (as occasionally, but very rarely happens) that the lithotrite fails to grasp or retain them? I think there is no doubt about the answer—viz., that it is the supra-pubic and not the lateral position." And again: "Finally, I think I am quite justified in believing that unless the operator has had a large experience of lithotrity (and there are not many of whom this can be affirmed), the high operation would generally be a safer proceeding than crushing for a calculus which is hard and much above an ounce in weight." In regard to the danger of impotence from peritoneal section, to which I alluded in my paper above mentioned, I am permitted to quote the following extract from a private letter received from my friend, Dr. A. H. Haemstadt, of Pottsville, Pa., a lithotomist of large experience: "Your eleventh accident—impotence—is well put; only one of eighteen whom I cut as boys, grown to manhood and married, has had issue as far as I can learn."

One of the strongest arguments in favor of the supra-pubic operation is that it avoids all interference with the *genital* organs, thus preventing the so frequent reflex disturbance of the nervous system accompanying the introduction of instruments through the urethra—a practical point to which, I believe, I was the first to publicly invite attention.—*London Lancet.*

